# Contact Lens Materials: A Materials Science Perspective

**DOI:** 10.3390/ma12020261

**Published:** 2019-01-14

**Authors:** Christopher Stephen Andrew Musgrave, Fengzhou Fang

**Affiliations:** 1 Centre of MicroNano Manufacturing Technology (MNMT-Dublin), University College Dublin, D14 YH57 Dublin, Ireland; christopher.musgrave@ucd.ie; 2 State Key Laboratory of Precision Measuring Technology and Instruments, Centre of MicroNano Manufacturing Technology (MNMT), Tianjin University, Tianjin 300072, China

**Keywords:** contact lens, materials, biomedical implant

## Abstract

More is demanded from ophthalmic treatments using contact lenses, which are currently used by over 125 million people around the world. Improving the material of contact lenses (CLs) is a now rapidly evolving discipline. These materials are developing alongside the advances made in related biomaterials for applications such as drug delivery. Contact lens materials are typically based on polymer- or silicone-hydrogel, with additional manufacturing technologies employed to produce the final lens. These processes are simply not enough to meet the increasing demands from CLs and the ever-increasing number of contact lens (CL) users. This review provides an advanced perspective on contact lens materials, with an emphasis on materials science employed in developing new CLs. The future trends for CL materials are to graft, incapsulate, or modify the classic CL material structure to provide new or improved functionality. In this paper, we discuss some of the fundamental material properties, present an outlook from related emerging biomaterials, and provide viewpoints of precision manufacturing in CL development.

## 1. Introduction

The market for contact lenses (CLs) is ever-growing, with over 125 million consumers as of 2004 [1], and an estimated global market size worth $7.1 billion in 2015 [2]. A quick search of patent literature shows that over 100 patents have been filed since 2000, representing the growing popularity of CLs. The applications of CLs range from corrective vision and therapeutics to cosmetic appearance [3,4]. Within these applications comes the demands from the end user of the lenses, including length of wear, comfort, durability, practically of handling, stability of vision, etc. This also means that within the applications of contact lenses comes the demands from manufacturers, such as material costs, ease of production, and reliability of the CLs, etc. Finally, the demands from manufacturers determine the parameters of the material which scientists must focus their research on for developing CL materials. This premise has guided materials scientists, from the creation of glass scleral lenses in the 1930s to rigid, non-gas-permeable polymethyl methacrylate (PMMA) in the 1940s. The 1960s and 70s ushered in hydrogel (polymer and silicone) lenses, with silicone hydrogel proving to be the most dominant kind of CL material today.

**Figure 1:**
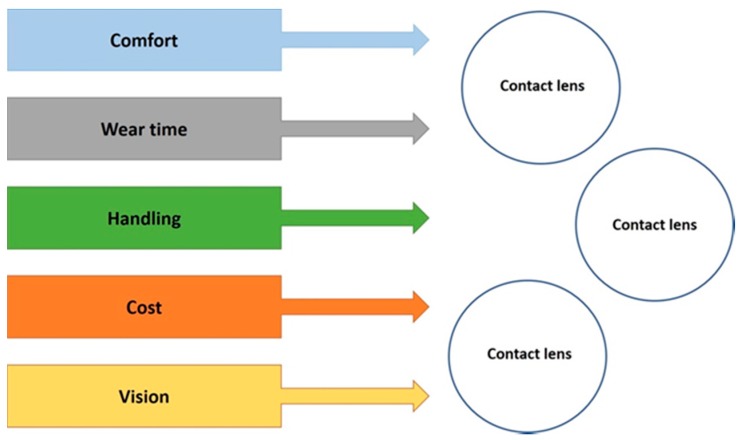
General user demands from CLs. Each parameter can be sub-divided into many categories, which is why the CL market is so vastly populated.

Commonly, the labels of “hard” or “soft” are used as blanket definitions of CLs [3,4]. Hard CLs are rigid (durable), gas-permeable lenses, whereas soft contact lenses are made of flexible, high-water-content material. Hard lenses are often interchangeably referred to as rigid gas-permeable lenses (RGPs); however, this is not strictly true. The first PMMA lenses could be classified as hard, whereas modern RGP lenses are, in fact, more flexible, due to the incorporation of low-modulus components—hence, they are more rigid, rather than hard. Another defining characteristic is that PMMA hard lenses have no oxygen permeability, whereas RGP lenses are permeable. On the other hand, a soft contact lens (SCL) is a highly flexible, oxygen-permeable material with often high water-content. This flexibility means that SCLs fit the shape of a user’s eye much faster than a rigid lens. SCLs can be disposed daily, weekly, or monthly. These blanket definitions of CLs can hint at its material properties, though not for certain. There is often an overlap in materials used between hard and soft lenses, such as silicone hydrogels and RGPs. Although both use silicone materials, the difference lies in factors such as the gel network and water content. Derivatives therein can further diversify the range of possible CLs and their properties. The requirements for CLs are quite extensive, and there are a huge number of existing CLs on the market to reflect this; daily-disposable lenses, weekly/monthly lenses, special fitting lenses, and even lenses that can be worn overnight. Users’ demands can be described by several general parameters, such as comfort, wear time, handling (cleaning, ease of use), cost, and vision specifications (Figure 1) [3,4].

PMMA is a durable, optically transparent polymer with limited hydrophilic character. However, PMMA has negligible oxygen permeability, which may lead to several eye health issues, such as hypoxia [5,6]. Researchers quickly discovered polymer hydrogels, which were typically based on hydroxy ethyl methacrylate (HEMA) [7,8]. These polymer hydrogels were composed of hydrophilic monomers, meaning they contained electrochemical polarity allowing interaction with water. This provided much greater biocompatibility than PMMA. These hydrogels were also an oxygen-permeable and flexible class of material that could hold a large percentage of water within the polymer network. These factors improved the comfort, oxygen permeability, and wear time of CLs, which resulted in many CL derivatives based on HEMA [9,10]. Progress did not stop, as the oxygen permeability of HEMA hydrogels was not sufficient for extended CL wear (>24 h) [3,4]. The next evolution of CLs was silicone-based rigid lenses and hydrogels. These materials have very high gas-permeability, meaning extended-wear lenses could be fabricated. However, silicone materials are inherently hydrophobic, meaning they are not comfortable due to factors such as poor wetting and abrasiveness. Polymer scientists overcame this by using copolymerization of a silicone monomer (e.g., siloxymethacrylates and fluoromethacrylates) with hydrophilic comonomers to add the desired hydrophilic character [11,12,13,14]. This was particularly successful for silicone hydrogel lenses, which is the most dominant CL material on the market today (64% in the US) [15]. Newer CL materials appeared in the 1990s, such as polyvinyl alcohol (PVA), which is a low-cost and very hydrophilic polymer [16,17]. In fact, the PVA hydrogel was the subject of investigation in the early 1990s as a potential CL material [18]. In addition, new surface coatings, such as polyethylene glycol (PEG) have appeared in recent years to improve the hydrophilicity of silicone-based CLs [19]. These are just two examples amongst a vast pool of relevant literature.

Despite this significant progress, more is demanded from CLs today. CLs provide a route for improving the quality of life. This could simply be for cosmetic reasons or for corrective vision in place of traditional spectacles, which remain the two most common uses of CLs today [20]. Cosmetic CLs can include anything from pigmented to prosthetic CLs. However, CLs are increasingly considered a platform for more proactive ophthalmic treatments. As the number of people developing myopia, glaucoma, and other eye conditions is increasing globally, more effective treatments are required [21]. One review paper estimated that over 1400 million people worldwide are currently suffering from myopia [22]. Myopia control using CLs has been a subject of debate [23]; however, there is mounting research that specialty lenses can control the progression of myopia [24]. CLs can offer one such treatment route for glaucoma; the drug-loaded lens can simply be placed onto the eye, and the drug is released onto the eye [25,26,27]. This is currently a hot topic, and has been the subject of several excellent review papers [27,28,29]. Therefore, it is clear that new and improved CL materials are required to deliver more effective treatments for these growing issues.

Many sources detail the general synthesis of hydrogels or discuss their end application, such as bioavailability, eye-fitting, CLs in practice, manufacturing, and much more [3,4,30,31]. There have been some articles that have talked specifically about materials for contact lenses, which provided important insights at the time [32,33]. Other sources have discussed general bioavailable materials [34,35] and the properties of CL brands [33], with a lot of modern research being concerned about drug delivery using CLs [27,36,37,38]. This review brings together research specifically about the latest development of CL materials, and touches on details from a materials science perspective. We aim to bring together the innovations in CL materials and explain the impact they have. We discuss general polymerization mechanisms and monomers used to produce CL materials, which are all considerations for designing new CLs. We then discuss new evolutions to the main classes of CL materials, such as RGP lenses, HEMA- and silicone-hydrogels, and their future perspectives. Finally, we highlight some particularly impactful materials, emerging materials’ technologies, and future manufacturing viewpoints.

## 2. Contact Lens Materials

### 2.1. Overview

**Figure 2:**
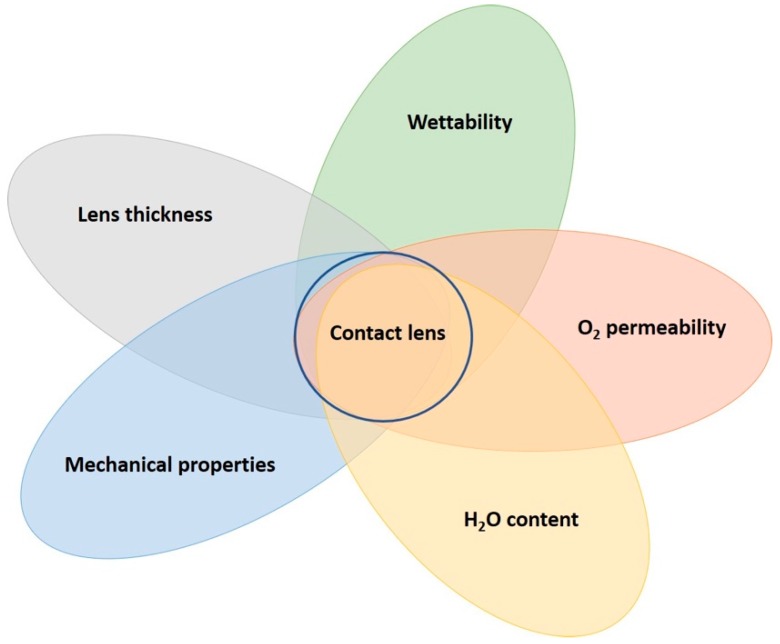
A CL lens is dependent on many parameters from a material science perspective. Stronger emphasis on specific characteristics are required, depending on the specific demand placed on the CL. The final CL material accounts for wear time and comfort. These characteristics are often dependent on the materials, but also includes manufacturing processes, such as plasma treatment.

To manufacture CLs, there must be a suitable polymeric material. This opens an incredible number of possibilities, not only from the range of polymers but to the formula of components within a given recipe. In addition, there can be considerations for the different types of polymerization mechanisms to form the same polymer, such as radical vs. catalytic polymerizations and derivatives. Within this, the polymerization conditions (temperature, initiator type, vessel used, etc.) can be altered to produce the same polymer but with different properties. Finally, the material must be suitable for the manufacturing stages, which include the synthesis, inspection, and packaging processes. The manufacturing stages have their own intricacies, which will be discussed later; thus, it is easy to quickly get lost in the search for the most suitable CL material. The extent of variation in materials is why there is such a wide range of CLs available today, and this has been extensively researched. Figure 2 contains some of the most important factors from a materials science perspective when designing CLs. From this, an assessment of current CL materials’ pros and cons is given in Table 1.

**Figure 3:**
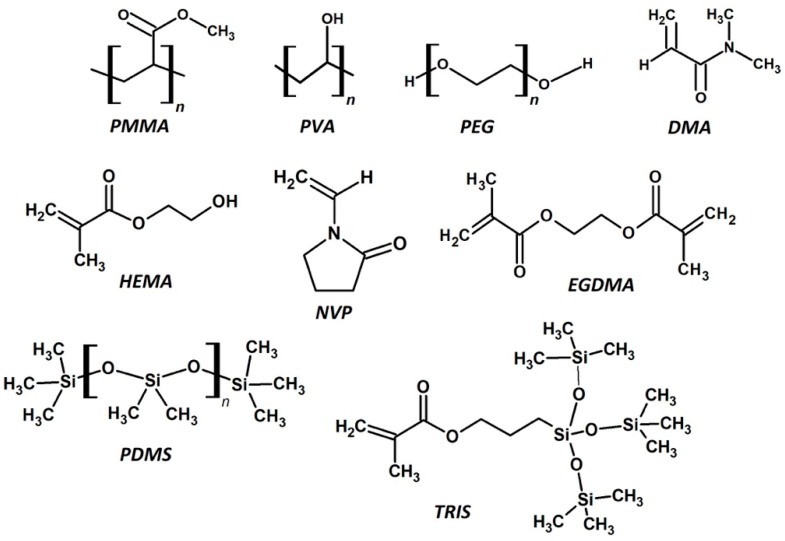
The chemical structures of common monomers and polymers used to produce CLs. This includes some macromonomers and cross-linking agents. PMMA—poly methyl methacrylate, PVA—poly vinyl alcohol, PEG—poly ethylene glycol, DMA—dimethyl methacrylate, HEMA—hydroxy ethyl methacrylate, NVP—N-vinyl pyrrolidone, EGDMA—ethylene glycol dimethacrylate, PDMS—poly dimethyl siloxane, TRIS—3-[tris(trimethylsiloxy)silyl]propyl methacrylate.

For clarity, a polymer is a large macromolecule composed of hundreds or thousands of a repeating molecule, called a monomer. Each monomer has a covalent bond between each connecting unit. Some common monomers and polymers used in the production of CLs can be seen in Figure 3. The process by which the polymer is formed is called polymerization. Another reactive molecule, the initiator, is used to begin the polymerization. Polymerization initiators are typically selected based on the interaction with the reactive functional groups within the monomers. Radicals are formed on the breakdown of the initiator, which then induce the polymerization by stripping a radical from the monomer functional group. Now the monomer has a free radical within itself, which reacts with a neighboring monomer functional group by stripping a radical from this group. The original monomer would have then formed a new bond with the new monomer unit, which now has a new radical to continue polymerizing. This propagation step is continuously repeated, thus forming the polymer over time, which extinguishes when there are no more monomers to consume. The polymerization terminates when the radical is quenched in another manner, such as by a radical scavenger. The initiator can be chosen for their practicality, such as ultra-violet (UV) or thermal initiators, which is a huge consideration for the manufacturing method in producing CLs.

### 2.2. Polymerization Mechanisms

Free-radical polymerization (FRP) is a type of chain-growth polymerization mechanism. Typically, free-radical polymerizations are often not easily controlled, producing high dispersity (large variations in molecular weights) of the resulting polymers. This can lead to a distribution of the polymer properties. High strain and tensile modulus polymers are often determined by the length of the polymer chains [39]. One advantage of FRP is that it is easy to form gelling networks with, as the polymerization has many initiation sites within the vessel. This allows for the simultaneous growth of many chains which can physically entangle or cross-link to form the gel network. Cross-linking is dependent on one of the monomers containing two functional groups, which enables chemical bonding to two different polymer chains. Cross-linking polymer chains often improves gelling and increases the modulus of the material. To date, most CLs are produced using free-radical polymerization [11,13,40,41,42,43,44]. It is facile and doesn’t require expensive reagents, such as catalysts. Furthermore, unwanted/unreacted chemicals can be removed from the material on post-fabrication cleaning processes. Catalysts are often composed of heavy metals, which is something to avoid for human health.

Today, most CLs are produced from a polymerization of two or more monomers [42,45]. Copolymers incorporate the properties of the individual polymers; consequently, copolymerization is often the first method used in overcoming issues with a single polymer. This principle has governed the development of CLs for many years. For example, silicone polymers are very hydrophobic, despite their high oxygen permeability. Therefore, they are not ideal as a homopolymer CL material. However, copolymers of silicones with a hydrophilic (highly polar) monomer can solve this problem. Copolymerization may also be used to enhance physical properties through the cross-linking of polymer chains by adding molecular weight to the chain. In some cases, adding a soft polymer can reduce the modulus of particularly tough materials (often silicone-based materials). Hydrophobic comonomers add oxygen permeability to materials that require improvement to this property, and silicone-based monomers are often used for this purpose. Table 2 contains the properties of common CL materials and related copolymers, including oxygen permeability, water content, moduli, and wear time. Wear time is from the perspective of the maximum wear time of a CL before eye health issues arise. More exhaustive resources comparing these properties are given elsewhere [3,4]. The effect of copolymerization is particularly noticeable for rigid materials derived from PMMA with a large reduction in the modulus, whereas the hydrogel classes of lenses have a much wider stable range of moduli, but large variations in water content and oxygen permeability.

**Figure 4:**
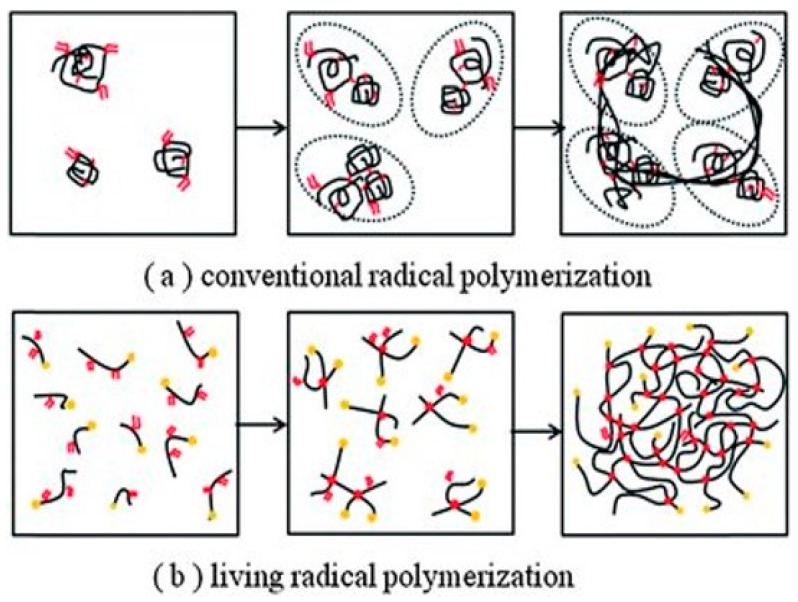
Schematic for the macromolecular structure differences between FRP and RAFT-polymerized materials. The RAFT polymer can be used to create a more ordered structure compared to FRP, which could be important to the macromolecular structure and final application of the material. Republished with the permission of The Royal Society of Chemistry, from Pushing the mechanical strength of PolyHIPEs up to the theoretical limit through living radical polymerization, Y. Luo, A-N. Wang, X. Gao, 8, 2012; permission conveyed through Copyright Clearance Center, Inc. [51].

Full-density polymers utilize molecular weight and intermolecular forces to provide the strength of the material; this is partly why PMMA has a high modulus. Other factors, such as the lens thickness of the final lens, is important too, whereas the properties of very low-density polymers (10% of full density, 90% air), such as porous materials are sensitive to small changes in cross-linking [46,47]. Another study demonstrated a reduction the Youngs modulus of up to 40% in a 90% porous material due to inefficient cross-linking [48]. This could be relevant to very high-water-content hydrogels on hydration from the final material properties and manufacturing considerations [49]. In fact, Maldonado-Codina and Efron highlighted the need for improving processes between polymerization batches and manufacturing methods. Other factors that are important include homogeneity of the wall vertices, uniform pore size, etc.; all of which are not guaranteed to be consistent with FRP. Moreover, if we consider SCLs to be porous cellular solids, these factors must be considered in future designs [50]. Although FRP has been the workhorse for fabricating excellent CL materials, they may not be producing entirely efficient networks for functional materials [51]. This could be another reason why there are no drug-delivery CLs on the market today, in addition to the many reasons stated by Dixon et al. [52]. Alternative polymerization mechanisms could be of interest to improve the physical properties of CL materials. Other kinds of methods include catalysts or controlled radical polymerization. One controlled method for radical polymerization is chain-transfer polymerization. This has been a popular area of growth in polymer science, including examining the potential in other bio-applications [53,54,55]. The chain-transfer agent accurately mediates the growth of the polymer chain, so that the molecular weight can be pre-designed [39,56,57]. This results in a low-dispersity polymer, meaning the resulting properties and structure are more reliable than FRP (Figure 4). There are many examples of hydrogels produced using chain-transfer agents [58,59,60]. One specific example of reverse addition fragmentation chain transfer (RAFT) polymerization encompasses silicone-based polymers [61]. Other researchers also used RAFT to modify polyacrylic acid pH-responsive hydrogels for drug delivery [60]. This opens the potential of RAFT-synthesized silicone- and conventional hydrogels as CLs. Recently, Zhang et al. synthesized a promising RAFT-polymerized soft contact lens based on polyallyl methacrylate and PEG components [62]. The lenses had low contact angles (<80°), high Dk values (>100 barrers), and elastic moduli ranging from 0.5–1.5 MPa, which are in the range of the CL parameters given in Table 1.

### 2.3. PMMA and Rigid Contact Lenses

#### 2.3.1. PMMA

**Figure 5:**
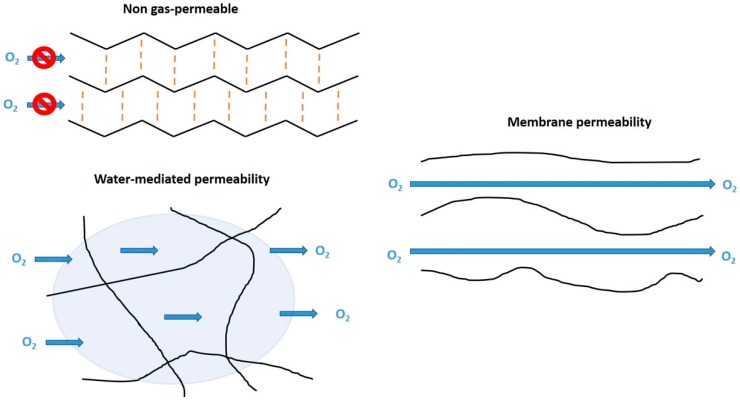
Schematic for gas-permeability mechanisms of CL materials from the perspective of polymer chains. These 2D models represent how oxygen passes (or not) through the molecular structure of the CL material. These schematics do not show factors such as extensive cross-linking or the macromolecular structure that would be present in a 3D structure.

Today, PMMA CLs occupy a market share of about 1% [15]; however, they are a useful place to begin to appreciate CLs materials—the properties of polymers that are suitable for ocular wear. One of the biggest issues with PMMA is that it has little to no oxygen permeability. This is due to the lack of mobility of polymer chains preventing the flow of oxygen or internal water to mediate the flow of O_2_ (Figure 5). This occurs in PMMA due to intermolecular forces, such as dipole–dipole bonding and physical entanglement that is prevalent between polymer chains. The dipoles are created by the negatively charged (electrochemical negative) oxygen compared with the adjacent positively charged (electrochemical positive) carbon and hydrogen atoms. Therefore, neighboring polymer chains can attract each other to provide thermodynamic stability to the polymer. These intermolecular forces also mean that PMMA has low free volume (space between polymer chains), meaning the chains do not rotate or move easily. In addition, PMMA does not contain large pendant chains that prevent the interaction of neighboring chains. All these factors together prevent the flow of oxygen through the polymer. However, functionalization of the PMMA surface can improve the hydrophilicity [63], which would be useful to these CLs.

**Figure 6:**
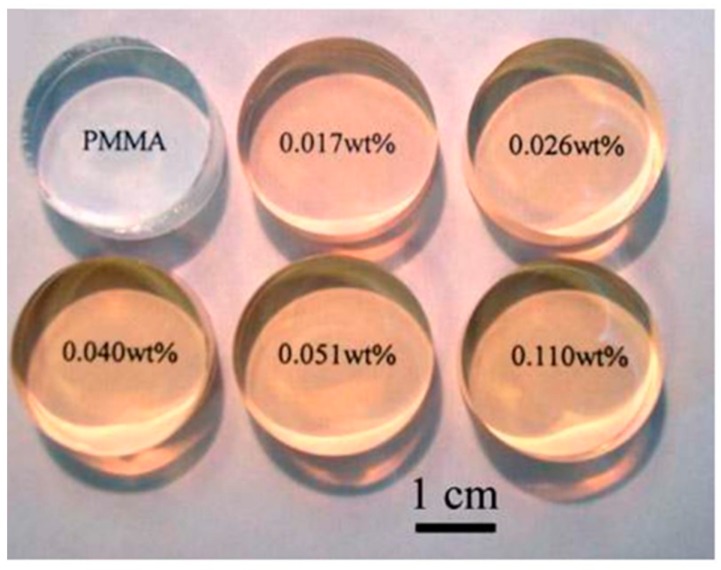
PMMA buttons loaded with ZnO quantum dots. The fabricated buttons would be suitable for lathe-cutting manufacturing to form a final CL. Reprinted (and adapted) with permission from John-Wiley and Sons [69].

In the recent literature, PMMA has typically been utilized as a reference material for investigating various effects of CLs on the eye [64,65,66,67]. These works are some of many with a particular focus on the effect of the PMMA lens on eye conditions, such as astigmatism [64], strabismus [68], and blepharoptosis [65]. Aliό et al. fitted patients who had received post-corneal refractive surgery with a variety of lens types, with RGP lenses showing the best results [64]. An interesting work by Li et al. showed fabrication of a possible new PMMA hybrid lens with zinc oxide quantum dots to reduce UV exposure [69]. Very low loadings of ZnO quantum dots (0.017 wt %) reduced the transmittance of UV light by 50%, yet retained suitable optical transparency expected of a contact lens (Figure 6). Perhaps the most novel advancement for PMMA lenses was the possibility of developing nanophotonic lenses [70,71]. Acid- and hydroxyl-functionalized fullerenes (C_60_) were attached to PMMA to try to harness the photo- and electro-activity of the fullerene. However, these nanophotonic lenses have now been a focus of SCL materials instead [72].

However, all may not be bad for PMMA, as van der Worp discussed the relevance of PMMA as scleral lenses [73]. In addition, PEG grafted onto PMMA is seen as a prosthetic eye replacement [74]. Overall, these are only a few works amongst an enormous amount of literature on contact lenses. The fact remains that PMMA is a very well-studied polymer; as such, the interest in developing new materials derived from PMMA is not exciting at present. Simply, the lack of many publications involving PMMA in the modern literature is strong evidence that PMMA is not relevant in today’s climate for CLs. The only hope is that PMMA can continue to be used for fundamental understanding, along with the factors that affect the eye gained when using PMMA as a reference CL material. For this reason, PMMA will remain a marginalized contact lens material for both research and commercial purposes.

#### 2.3.2. Other Rigid CLs

Modern rigid gas-permeable (RGP) lenses have moved away from PMMA, or significantly reduced the molar fraction of PMMA in many copolymers. Efron wrote an obituary for RGP lenses stating the reasons why these lenses are obsolete [75]. Nevertheless, RGP lenses (including PMMA) still account for about 14% of all lenses fit in the US as of 2017 [15]. Modern research concerning RGP lenses is in regard to their application, treatment, and effect on eye conditions, rather than innovations on the material [76,77,78]. Eggnik et al. reported that a large-diameter RGP lens of unknown composition was used to improve post-surgery treatment for laser in situ keratomileusis (LASIK) patients [79]. The success of RGP lenses lies in their rigidity, causing a reshaping of the cornea, which was useful for some post-surgery treatment [80]. Soft lenses are sometimes unsuitable for treatment as they are very malleable, and therefore will shape to the user’s eye. These other works often use commercially manufactured lenses to carry out their research; therefore, it is more difficult to know the exact composition and methods by which these materials were synthesized. However, it is known that the typical composition of these lenses are based on fluoro silicone or siloxane (such as PDMS and TRIS) acrylate moieties, alongside hydrophilic monomers such as HEMA, NVP, and methacrylic acid (MAA) [4,76]. Baucsh & Lomb were assigned a patent for polysiloxane, copolymerized with urea moieties prepolymers to from RGP lenses [81]. The urea (or carbamides (CO(NH_2_)_2_) moieties added hydrophilic properties. These vinyl end-capped prepolymers were then copolymerized with well-known monomers such as NVP, MAA, MMA, and TRIS to form the RGP lens.

Although Efron’s prediction may not have come to pass, what is true is that there is a lack of material innovation for RGP lenses. This simply could be due to the fact that RGP lenses are unfashionable from a research perspective, particularly with the booming field of hydrogels. In fact, Efron first alludes to this by commenting on the marketing for SCLs over RGP lenses, and that the lack of finance directed at improving these materials has caused their decline. Smart or wearable biosensors show promise for RGP lens materials [82], but even this field is being dominated by SCL materials [83]. Perhaps the route to reinvigorating RGP lenses is through the growth of augmented reality (AR) technology. Patents for the design of an AR contact lens have been assigned to companies such as Samsung and RaayonNova in 2016 and 2017, respectively [84,85]. However, it is likely that the first lenses will borrow heavily from existing RGP lens materials. This is in contrast to other smart CLs, which are often based on SCL materials [86].

### 2.4. HEMA-Derived Hydrogels

**Figure 7:**
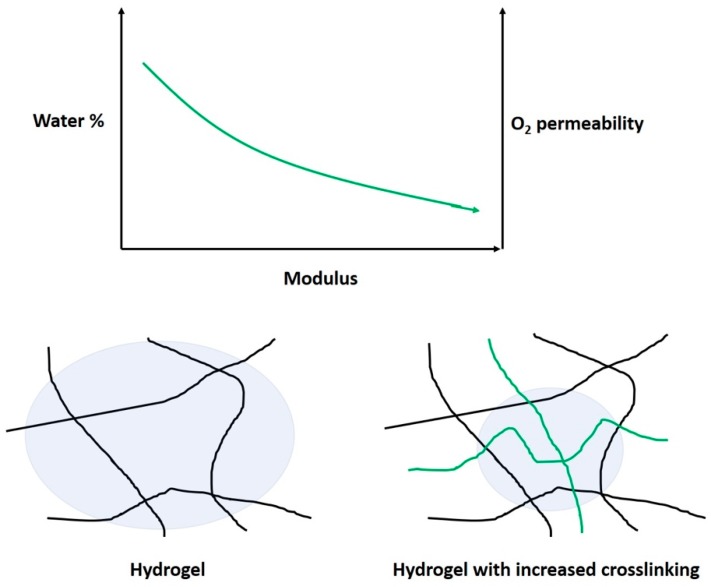
Schematic on the effect of cross-linking on the modulus, water-content percentage, and oxygen permeability. The increased cross-linking prevents (green) the polymer chains from swelling, compared with lower-modulus gels.

HEMA and related hydrogels are a high-water content, oxygen-permeable polymeric material. These hydrogels can have water content between 20–80% depending on the comonomers, with a hydrogel composed of only HEMA containing about 38% water. HEMA’s highly polar properties mean these CLs have generally suitable wetting properties, meaning they are comfortable. The oxygen permeability of these gels is suitable for longer wear, but not to the same extent of silicone-based lenses. HEMA-derived hydrogels form an important part of the market, occupying about 22% [15]. HEMA is commonly copolymerized with monomers such as EGDMA, MAA, and NVP. NVP and MAA increase the water content of hydrogels due the strong hydrophilic character arising from amine, carboxylic acid, hydroxyl groups, etc. Therefore, these comonomers also influence the wettability of the surface [87,88]. The mechanical properties can be improved by using a cross-linking molecule, such as ethylene glycol dimethacrylate (EGDMA). EGDMA has two functional groups allowing the formation of covalent bonds between two individual polymer chains. This increases the mass of the polymer dramatically, and improves its ability to form a gel network. However, the crosslinks also reduce the polymer-chain motion, which can be a factor in swelling and oxygen transport [88]. The water content and cross-linking affect the modulus and oxygen permeability of the hydrogels (Figure 7). As such, a balance must be reached between these parameters when designing a CL for a particular application.

To improve CL materials, it is vital to better understand the properties of these hydrogels. The properties of HEMA-based hydrogels were shown to vary as a function of use by patients [89]. Tranoudis et al. measured the properties of lenses, such as total diameter, oxygen permeability, and the back optic zone radius, at temperatures ranging from 20–35° and before/after 6 h of wear. All the materials were altered with statistical significance; however, according to the summary tables, HEMA-VP 70% (meaning 70% water content) was moderately stable. In another study, the authors showed that free-to-bound water content was an important factor for the rate of dehydration and oxygen transport in SCLs [90]. The bound water to the polymer may be a factor in stability of hydrogels, such as HEMA-VP 70%, compared with other gels. Tranoudis et al. published another paper to more accurately characterize the tensile properties of the same organic hydrogels [91]. They concluded that hydrogels with high water content did not necessarily correlate with poor mechanical properties. This is largely true, and is an uncomplicated way of summarizing the data. However, comparing different polymer systems can be difficult due to the different intermolecular forces that exist to provide stability (or lack of) based on different chemical compositions. There are properties of the polymer structure which could reveal more factors that affect the mechanical properties of hydrogels. Explanations by Ashby may reveal the reasons for inconsistent properties between batches of the same polymeric material [50]. Porous cellular solids with varying wall thicknesses and lengths between vertices are some of the factors that influence the mechanical properties of porous materials. Therefore, it is important to study the polymer’s physical structure and polymerization mechanisms. Use of ^1^H Nuclear Magnetic Resonance (NMR) relaxation times by Woźniak-Braszak et al. was a novel characterization method used to probe the dynamics of free water in contact lenses [92], where they showed an increase in free water in four-week-old lenses compared with new lenses (83% to 71%, respectively). These studies together provide a more detailed understanding of hydrogels, which in turn can be used for the better design of CLs.

Currently, modification of the HEMA-hydrogel structure is paving the way for new lenses, innovations, and applications. Protein deposition on the eye remains a concern, particularly for SCL materials based on HEMA [93,94]. Common comonomers, such as MAA and NVP, increase the protein deposition. Both comonomers introduce hydrophilicity and electrochemical charge to the hydrogel, which attracts the proteins from the tear film [93]. Lord et al. also showed that the protein uptake could affect the water content of a HEMA-MAA hydrogel. This could be related to observations by Tranoudis et al. where a HEMA-MAA hydrogel lost 15% water content across a 6 h period [89]. An interesting report by Borazjani et al. regarding the bacteria adhesion of *Pseudomonas aeruginosa*, a risk factor in keratitis, to a HEMA-hydrogel was no less than that of a silicone hydrogel [95]. Borazjani et al. also reported that adhesion of the bacteria was more related to the strain of bacteria than the lens material. Another study by Szczotka-Flynn et al. demonstrated that biofilms of bacteria (particularly *Pseudomonas aeruginosa*) were resistant to the antimicrobial action of CL solutions [96]. Dutta et al. looked into the literature of this area by considering the material type, length of wear, and bacterial strains on the cumulation of the bacteria on contact lenses [97]. New strategies to prevent this have been in development since the early 2000s, including further modification of the lens material. A more detailed and extensive article on antimicrobial strategies was published by Xiao et al. [98].

Grafting additional substances to HEMA has proven to be an effective method of modification for improved lens functionality [99,100,101]. Some specific examples include grafts to reduce the protein deposition or increase the anti-microbial action of contact lenses. A successful product based on a HEMA hydrogel lens incorporated with silver nanoparticles (AgNPs) has been on the market in the UK (MicroBlock^®^). Other researchers looked at the impact of the monomer composition of the HEMA hydrogel contact lens on AgNP uptake and performance [102]. They noticed that HEMA-MAA-EGDMA gels had the strongest affinity to the nanoparticles, soaking in AgNPs from 10 to 20 ppm. Other interesting antimicrobial graft materials include melimine and polymyxin B. Melimine is a synthesized antimicrobial peptide consisting of 29 amino acid units, and was covalently bonded to HEMA-MAA-EGDMA using an acidic (pH = 5) buffer to induce the reaction to the pendant acid groups on the lens [103]. This post-hydrogel modification may not be entirely practical for manufacturing, but is interesting due to the excellent properties of the resulting hydrogels. Polymyxin B is another anti-microbial macromolecule, and was grafted using an azobisisobutyronitrile (AIBN) free-radical initiator during hydrogel synthesis [104]. The action of polymyxin B induces greater water permeability of bacterial cells, eventually leading to bursting through the increased water uptake. Sato et al. modified HEMA by copolymerization with moieties such as 2-methacryloxy ethyl phosphate (MOEP) to facilitate drug delivery [105]. The MOEP adds additional anionic character, which was used to bind a model drug to the hydrogel. The drug release profile was a superior HEMA-MAA hydrogel due the stronger ionic group based on the chemical changes with pH.

**Figure 8:**
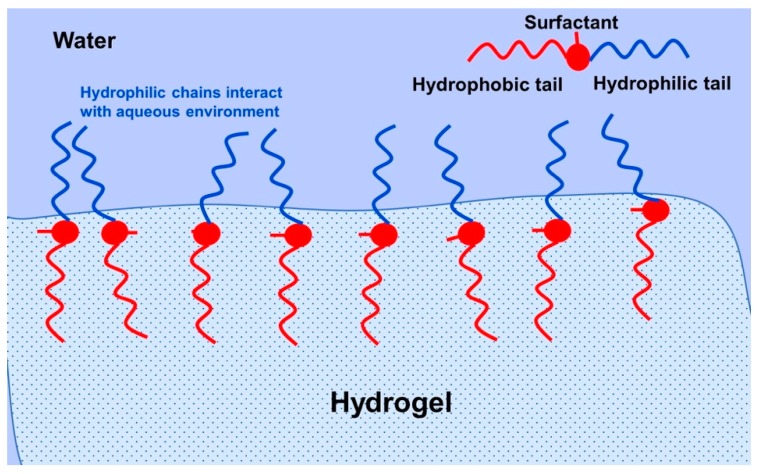
Schematic for the interaction between the aqueous tear film and a surfactant bonded with a hydrogel lens. Reprinted from Journal of Colloid and Interface Science, 445, Bengani, L.C.; Scheiffele, G.W.; Chauhan, A.; Incorporation of polymerizable surfactants in hydroxyethyl methacrylate lenses for improving wettability and lubricity, 60, Copyright 2014, with permission from Elsevier [106].

**Figure 9:**
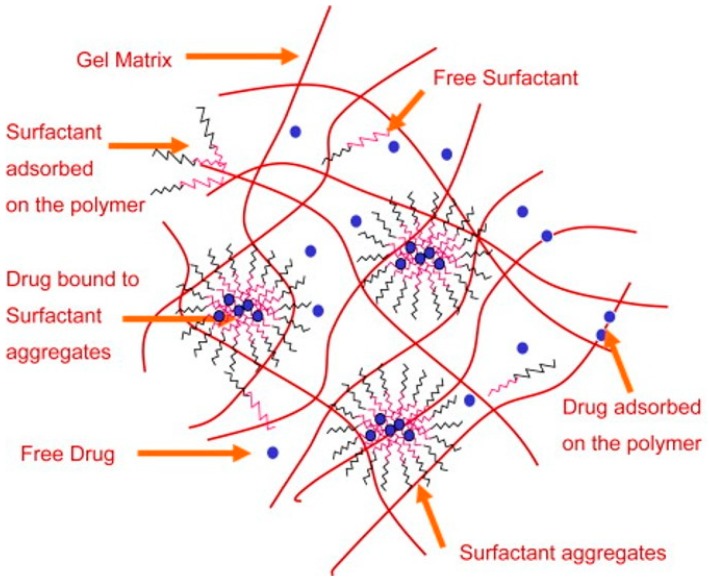
Drug-loaded hydrogel loaded with surfactants. The aggregation and leaching of the surfactant acts as a drug delivery vehicle. Reprinted from Biomaterials, 30, Kapoor, Y.; Thomas, J.C.; Tan, G.; John, V.T.; Chauhan, A. Surfactant-laden soft contact lenses for extended delivery of ophthalmic drugs, 867, Copyright 2008, with permission from Elsevier [110].

Other modification materials include the incorporation or grafting of surfactants. A surfactant consists of a hydrophobic and hydrophilic component, and is primarily used to promote the reduction in surface tension between two immiscible liquids. Bengani et al. employed the novel use of polymerizable surfactants attached to HEMA hydrogels to enhance wettability and lubricating properties [106]. They achieved a 10° reduction in water-contact angle with about 2.4 wt % surfactant, which was covalently bonded to the hydrogel by UV polymerization. The low-surfactant loading meant that the HEMA-hydrogels remained below 45% water content, which indicates the power of such a technique. In this case, the hydrophilic component interacts with the aqueous tear film and the hydrophobic part remains in the hydrogel (Figure 8). There is much modern literature that still reports high rates of contact lens discontinuation due to discomfort and dryness [107,108,109]. This emphasizes the importance of new techniques, such as surfactant loading/grafting employed by Bengani et al. Other uses of surfactants include use as a drug delivery system for cyclosporine A in modified HEMA-hydrogels [110]. In this case, the surfactant leached out of the hydrogel with the drug encapsulated within surfactant aggregates (Figure 9). This is now becoming a more researched topic, using a larger number of surfactants to deliver drugs [111,112,113,114]. These works have used a diverse range of surfactants, from cationic to non-ionic, with molecular weights ranging from about 400–12,500 g mol^−1^. This wide variation in surfactant properties is interesting, suggesting that the hydrogel structure is highly adaptable to such modifications.

In summary, HEMA-hydrogel CLs are in an exciting place. The ability to design new materials is due to the flexible hydrogel structure composed from many functional groups. Essentially, the core of the hydrogel has remained relatively unchanged, due to the principal properties that matter to CLs, such as oxygen permeability, water content, wettability, etc. Modern HEMA hydrogel lenses have evolved through modification of the hydrogel structure through techniques such as encapsulation and grafting. Some of the materials used for modification were surfactants, nanoparticles, and anti-microbial agents, all of which are diverse in themselves. As such, the works presented here are only the beginning of an emerging field. There were also efforts to better understand the mechanical properties of the HEMA-hydrogels, such as dehydration rates, bound and free water content, and factors influencing protein absorption into the hydrogels. From a better understanding of hydrogel properties, new manufacturing processes can evolve to further improve the material specifications. This is encouraging progress for designing better materials, which will only keep evolving as more and more chemical modification techniques and materials are employed.

### 2.5. Silicone-Derived Hydrogels

Silicone-based hydrogels are the most common type of CL material today, occupying about 64% of the US market [15]. This includes silicone, silioxanes, fluorosiloxanes, and derivative materials. Their popularity is linked to the fact that these CLs have the highest oxygen permeability of all contact lens materials (>100 Dk, typically). Silicone CLs are often durable, originating from the high Si–O bonding energy, often with a higher modulus than conventional polymer hydrogels. This typically applies to RGP lenses, as the modern silicone hydrogel has a similar modulus to HEMA-derived hydrogels. The high modulus is linked to causing irritation to the eye, such as the conjunctiva of the inner eyelid [4]. In fact, the discomfort and dryness of silicone-based lenses are two of the main reasons for users’ discontinuation [107,109,115,116]. Specifically, the wettability of the lens and the incompatibility with the cornea environment in vivo requires detailed study to improve these parameters [117]. The patent literature contains much about improving the wettability of lenses, with companies such as CooperVision [118] and Johnson and Johnson [119] having filed for patents to develop such lenses. This includes using many combinations of comonomers with silicone monomers. However, similarly to the preceding sections, modern silicone lenses have not deviated from the base materials since they were first developed. Nowadays, new chemical techniques look to evolve the silicone CL material beyond its predecessors.

**Figure 10:**
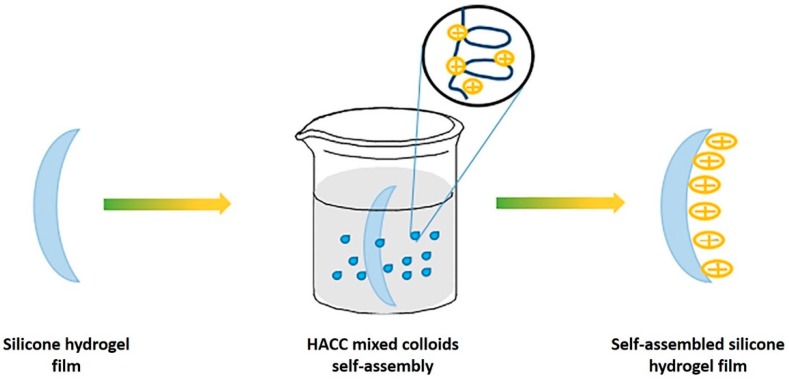
Schematic for the adhesion of a self-assembled layer onto the surface of silicone hydrogel. Reprinted (and adapted) from Colloids and Surfaces A, 558, Tian, L.; Wang, X.; Qi, J.; Yao, Q.; Oderinde, O.; Yao, C.; Song, W.; Shu, W.; Chen, P.; Wang, Y. Improvement of the surface wettability of silicone hydrogel films by self-assembled hydroxypropyltrimethyl ammonium chloride chitosan mixed colloids, 422, Copyright 2018, with permission from Elsevier [127].

Silicone hydrogels have commonly been subject to post-fabrication processes. Some, such as plasma treatment, are used to improve lens wettability by manufacturers [120,121,122]. This technique has been proven very effective; however, there are still a number of issues relating to silicone CLs [123]. Santos et al. suggested that protein absorption of commercial silicone hydrogel lenses was independent of the plasma treatment [124]. They summarized that the inherent hydrophobic character of the lenses helped in reducing the deposition. With this in mind, new chemical modification techniques, with or without plasma treatment, are becoming of interest. The use of hydrophilic and hydrophobic lipids by Bhamla et al. investigated the wetting and dewetting character of a Balafilicon A lens [125]. They showed that a hydrophobic lipid (meibum) could reduce the wetting area on the lens surface by nearly 70% in 50 s. Lin et al. modified the lens surface to adhere polyelectrolyte monolayers (PEMs) of chitosan and hyaluronic acid [126]. Chitosan is a naturally derived polymer (from chitin) with high bioavailability originating from the hydroxyl and amine groups within the structure, lending itself to lens modification. They modified the plasma-treated silicone surface to create negatively charged groups, which allowed the formation of the positively charged chitosan layer. Then, the negatively charged hyaluronic acid formed a layer on this surface to form alternative layers. The contact angle was reduced from 90° in the mother lens to up to 50° in the PEM-modified lenses. Similarly, a modified chitosan was also used successfully by Tian et al. to form a chitosan layer on the surface of the lens (Figure 10) [127]. Hydroxypropyl trimethyl ammonium chloride chitosan is a quaternary ammonium salt and is positively charged, which facilitates the formation of self-assembled layers. These pendant chemical groups could lead to further modification of the lens. Thissen et al. modified silicone lenses with a poly ethylene oxide (PEO) graft to the surface for anti-biofouling properties [128]. First, they used plasma polymerization to adhere allyl amine to the surface of the lens, and then used a reducing solution of sodium cyanoborohydrate and PEO to complete the PEO graft. A similar process using acrylic acid was performed by Dutta et al. to attach a melamine-derived peptide (Mel4) to the surface of a range of silicone lenses (lotrafilcon A, lotrafilcon B, somofilcon A, senofilcon A, and comfilcon A) [129]. They also used an acid buffer solution to covalently bond the Mel4 peptide to the surface of the lenses [130]. The peptide improved the anti-microbial properties of the lenses without causing other irritations to the tested subjects (rabbits).

Other materials were of interest as new grafting or encapsulation components into silicone-based hydrogels. Tuby et al. used Zn-CuO particles as an anti-microbial film on CLs composed of silicone hydrogels (Bausch&Lomb) [131]. They used a base bath (pH = 8) loaded with the nanoparticles to soak into the lenses to induce uptake of the nanoparticles by the hydrogel. They also commented that the nanoparticles did not leech easily from the structure, which would be an important safety concern. Jung et al. used a polymer nanoparticle (propoxylated glyceryl triacrylate) to deliver the drug timolol, which is used for glaucoma treatment [132]. The lenses also incorporated the nanoparticles through a soaking method, which was a phosphate buffer solution (pH ≈ 7.4). Although the nanoparticles did not affect optical transparency, they noted an undesirable decline in the water content, oxygen permeability, and modulus. Willis et al. grafted hydrophilic phosphorylcholine (PC) to the surface of silicone lenses using several Michael-type addition steps [133]. The coating reduced the water contact angle by nearly 50°, which was an impressive result. One concern they had was the lowering modulus by 0.5 MPa in the PC-treated lenses compared with unmodified lenses. Wang et al. used an interpenetrating network of silicone and PC to synthesize the hydrogel to achieve similar wettability results [134]. A 35–80 nm layer of PC was also grafted onto silicone using UV light (305 and 365 nm) to induce the polymerization graft [135]. The surface graft did not affect the oxygen permeability and modulus, but reduced the lysozyme and fibrinogen deposition and water contact angle. Wang et al. grafted polyethylene glycol methacrylate (PEGMA) to silicone using UV light again, and again the hydrogels retained excellent properties, such as >140 Dk and >1 MPa elastic modulus [136]. Synthesis of silicone hydrogels also incorporating PEGMA by Lin et al. successfully reduced the lysozyme and human serum albumin (HSA) deposition by 82% and 77%, respectively [45]. However, the increasing volumes of PEGMA affected other properties of the lens, such as increasing brittleness above 20% PEGMA content. The PEGMA polymer brush is a known anti-biofouling material [137,138]. Its low cost and easy incorporation is likely worth pursing for other CLs. These studies highlight the impact of surface modification techniques on improving CL properties. However, manufacturing aspects need to be considered to realize these modifications in a new generation of CLs.

In summary, silicone hydrogel lenses are targets for highly biocompatible coatings that can be grafted/encapsulated to the surface of, or within, the lenses by chemistry techniques. In these experiments, the performance of the lens was improved, particularly towards anti-biofouling applications. Hydrophilic anti-biofouling materials are being applied more often to silicone hydrogel CLs [139]. This emphasizes the innovations which silicone-based hydrogel lens materials are undergoing. For silicone hydrogel, there is a two-fold benefit of these anti-biofouling materials: (1) These hydrophilic grafts/modifications are the route to anti-biofouling behavior, and; (2) they are hydrophilic, increasing the wetting character of the lens. This effect is also relevant to HEMA-based lenses; however, it is more impactful to silicone lenses, given their inherent wettability disadvantages and market size. Continued improvements to these materials will result in continued market dominance for silicone-based hydrogel CLs.

### 2.6. Other Contact Lens Materials

#### 2.6.1. Polyvinyl Alcohol Hydrogels

PVA is a synthetic polymer that contains many hydroxy (–OH) groups, one in each repeating monomer unit. This is the source of PVA’s excellent hydrophilic and biocompatible properties [140]. As such, it is clear why this material is an interesting contact lens material. PVA hydrogels were of attention to several researchers in the early 1990s [17,18]. Even without modification, PVA CL hydrogels were also shown to have lower protein absorption rates than HEMA and MMA/VP hydrogels [18]. However, it wasn’t until the late 1990s that this material broke into the market in the form of the Nelfilcon A lens [16]. These lenses have a Dk of about 26 barrers and high wettability, which is acceptable for daily wear. Produced by CIBA vision, the PVA hydrogel was synthesized using water as the solvent and produced inside a transparent mold to allow UV initiation of the polymer solution. Water was an environmentally friendly choice of solvent which also did not hinder the polymerization stages. Buhler et al. formed the PVA hydrogel by adding a new functional group to facilitate cross-linking between chains [16]. In acidic conditions and a suitably reactive dialdehyde group, PVA can be functionalized with a stable cyclic-acetal group. This can then crosslink with other PVA chains to form a crosslinked hydrogel. More recently, PVA has been used as a tool for producing more comfortable lenses [141,142] or to facilitate the loading of a colored pigment into the lens [143]. One of the comfort mechanisms include elution of an unbound PVA polymer from the contact lens into the tear film. Non-crosslinked PVA with an approximately 47,000 molecular weight was soaked into a cross-linked PVA lens. The high molecular weight and bioavailability meant that PVA leeched out of the lens to lubricate the eye as a sort of artificial tear component [142,144]. Researchers identified that the higher molecular weight species took longer to elute from the lens than lower molecular weight species. Although these examples are strictly not using PVA hydrogels, they highlight the importance of this material for other ophthalmic applications. This even extends to areas such as corneal replacement [145].

**Figure 11:**
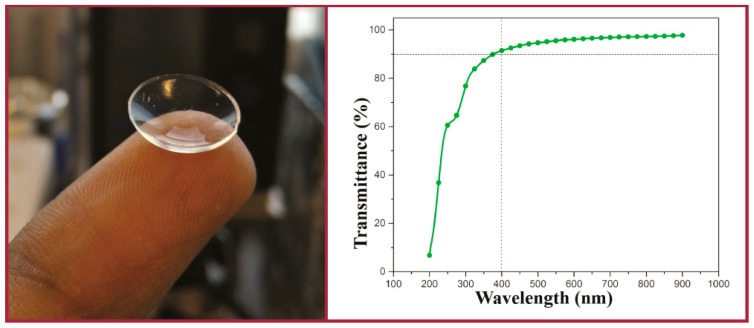
Optical image (**left**) and the transmittance data (**right**) for PVA hydrogels incorporating nanocellulose. Reprinted (adapted) with permission from Tummala, G.K.; Rojas, R.; Mihranyan, A.; Poly(vinyl alcohol) hydrogels reinforced with nanocellulose for ophthalmic applications: general characteristics and optical properties, Journal of Physical Chemistry B, 2016, 120, 13094. Copyright 2016 American Chemical Society [148].

There has been some investigation into the modification of PVA hydrogel CLs. Xu et al. modified the PVA structure with β-cyclodextrins (β-CDs) to improve drug-loading of puerarin and acetazolamide [146]. They functionalized both the β-CDs and PVA with a methacrylate moiety with *N*,*N*-dimethyl-4-pyridinamine and glycidyl methacrylate to achieve the necessary functionality. Then, both methacrylated species were copolymerized to form the hydrogel. This method also resulted in incorporation of 30 wt % of β-cyclodextrin. Sun et al. also used a functionalized β-CD polymer incorporated into a PVA film for the delivery of voriconazole [147]. A β-CD solution containing dissolved voriconazole was mixed with a PVA solution and then used to form electrospun nanofibers. PVA cross-linked with cellulose offers another a potential route to improved contact lens materials [148,149]. Cellulose is a naturally occurring polymer with enormous potential for bio-applications [150]. Mihranyan chemically bound the cellulose microcrystal “whiskers” onto the surface of PVA using a 2,2,6,6-tetramethyl-1-piperidine oxoammonium salt (TEMPO)-mediated surface oxidation [149]. The TEMPO technique is a special technique used for regioselective oxidation of cellulose to preserve crystallinity [151]. The technique only functionalizes the surface of the material. Although the physical properties of the PVA hydrogels improved, the opaque material would not be ideal as a CL material. Tummala et al. incorporated nanocellulose, a transparent version of cellulose, as nanofibrils or nanocrystals into a PVA hydrogel, improving the physical properties [148]. In addition, the lens retained excellent optical properties, with >90% transparency to visible light (Figure 11). They used a mixed solvent system of water:dimethyl sulfoxide (DMSO) at ratios between 60–80% DMSO content to facilitate the solvation of the nanocellulose components. These nanocellulose PVA hydrogels were investigated for their light-scattering properties by Tummala et al. in another work [152]. The hydrogel composition and angle of light affected the scattering of light in the range of 3% to 40%, with the nanocellulose-functionalized PVA hydrogel reducing the scattering. The nanocellulose hydrogels were then used to encapsulate acrylic-acid-functionalized chitosan nanoparticles as a vehicle for a lysozyme-triggered release of a model drug. Lysozyme (0.2 mM)-mediated hydrolysis of the chitosan-functionalized nanoparticle would trigger the release of the drug.

The PVA hydrogel is a relatively new class of hydrogel lens compared with silicone- and HEMA-hydrogels. The low cost, high bioavailability, and wettability properties mean PVA hydrogels will remain important to the CL industry. This means there is a lot of design space for investigation into modifying the structure to improve lens functionality. There are an increasing number of modification techniques and materials that have yet to be explored with PVA hydrogels. The secondary alcohol groups within the polymer chain are very easily modified for grafting, as shown by cellulose or β-cyclodextrin incorporation. As with HEMA- and silicone-hydrogels, the grafting or encapsulation of anti-microbial agents is just one of many interesting avenues of research that is possible for PVA hydrogels.

#### 2.6.2. Hyaluronic-Acid-Modified Hydrogels

**Figure 12:**
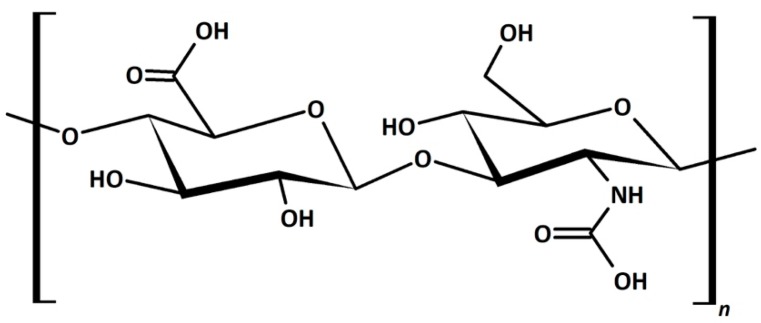
Chemical structure of hyaluronic acid. The repeating unit within the polymer is hydrophilic with many highly polar groups, such as the amine, acid, and hydroxy groups.

Hyaluronic acid (HA) is a hydroscopic biopolymer that naturally occurs within the human body. HA has already been mentioned in previous sections. Here, we explain why this material is significantly impacting CL research. This is an important material for a wide range of tissue-engineering purposes [153]. From the chemical structure, we can see why it is useful for incorporating into CL materials (Figure 12). The amino acid and hydroxyl groups present in each repeating unit provide the necessary hydrophilic character, leading to high biocompatibility. As such, this material has been of interest in developing ophthalmic treatments, such as lubricating solution [154] or contact lens modification [155]. The incorporation of HA was shown not to affect the surface morphology of the CL even after 12 h of wear, showing the stability of these modifications [156]. HA is typically a graft/encapsulating material to other established CL hydrogels. The commercial success of HA is apparent with the Bausch&Lomb Biotrue™ solution and Open30 (by Safilens) lenses incorporating HA as a lubricating agent. Other ophthalmic treatments and modifications include HA use as a treatment for dry eyes, [157,158] modification of the lens material [126,159,160], and drug-delivery mechanism [161,162,163].

**Figure 13:**
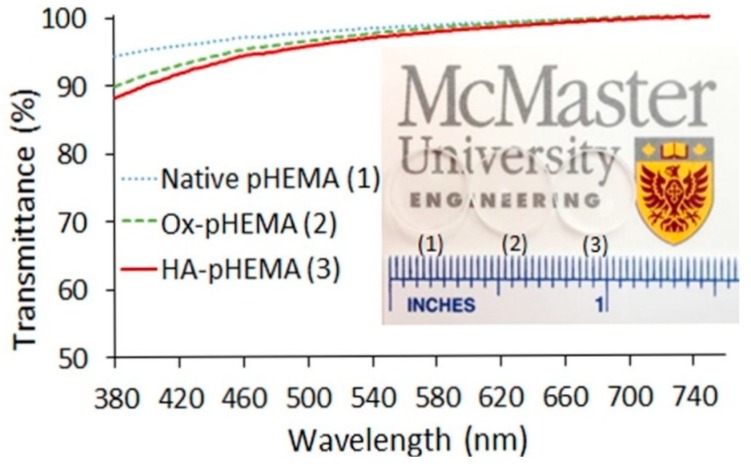
Optical properties of HA-HEMA, oxidized-HEMA (OX-HEMA), and unmodified HEMA lenses. The transparent hydrogels can be seen just above the ruler markings. Reprinted (adapted) with permission from Deng, X.; Korogiannaki, M.; Rastegari, B.; Zhang, J.; Chen, M.; Fu, Q.; Sheardown, H.; Filipe, C.D.M.; Hoare, T. “Click” chemistry-tethered hyaluronic acid-based contact lens coatings improve lens wettability and lower protein adsorption, ACS Applied Materials and Interfaces, 2016, 8, 22064. Copyright 2016 American Chemical Society [166].

HA was investigated as a reusable wetting agent, and considered as a vehicle for the delivery of timolol within a silicone hydrogel lens [164]. HA is hydroscopic, meaning it was used to add water content to a material without a significant reduction in the other hydrogel components of DMA and TRIS [160]. All of the silicone hydrogels Paterson et al. produced had a water contact angle of less than 50°, emphasizing the wettability qualities of HA. The wetting agent was a copolymer of PEG and HA, which was first copolymerized before being soaked into the HA-modified silicone hydrogels. Van Beek et al. crosslinked/physically entrapped HA into HEMA hydrogels by using 1-ethyl-3-(3-dimethylaminopropyl)-carbondiimide (EDC)/diaminobutane-4 dendimer to provide the necessary functionality for a reaction [159]. Furthermore, HA in very low amounts (5 g/L solution) reduced the water contact angle of the hydrogels by about 15–25°. These HA-modified hydrogels also showed less lysozyme absorption, which can often be a criticism of HEMA-derived hydrogels. However, large molecular weight (M_w_) HA (169 kDa) had reduced optical transparency between 500–800 nm versus the lower M_w_ HA (35 kDa). On the other hand, the 169 kDa HA was responsible for the lowest amount of protein deposition. Korogiannaki et al. covalently bonded HA to the surface of HEMA by using a nucleophilic Michael-addition thiol-ene “click” chemical reaction [165]. These lenses retained an optical transparency level of >92%, and decreased the water contact angle and rate of lens dehydration. A more detailed mechanism of the covalent bonding of HA to the HEMA hydrogel structure was presented by Deng et al. [166] “Click” chemistry was used with adipic acid dihydrazde (ADH) to anchor HA to HEMA. HEMA was first oxidized to provide the functionality for bonding the ADH-HA anchor. The lens properties virtually remained unchanged, such as the modulus and optical properties (Figure 13). They also measured a reduction in the rate of dehydration of the lens compared with the unmodified lenses. Weeks et al. used UV light to covalently bond a methacrylate modified HA to HEMA and silicone hydrogel lenses [167]. In all cases, the HA-modified hydrogels had a greatly reduced water-contact angle and reduction of lysozyme absorption. They also pointed out that HA with little or no methacrylation on the surface would not prevent the interaction of the protein with the surface of the hydrogel, whereas if HA was entrapped inside the hydrogel, the protein could not interact with the surface at as many points, preventing its deposition.

HA is an increasingly popular material for the modification of both HEMA-hydrogel and silicone-hydrogel CLs. The high bioavailability and straightforward incorporation methods have already seen recent commercial success for HA. In these cases, HA was a lubricating agent rather than any physical/chemical modification of the lens material. Recent works regarding HA are exciting, demonstrating the ability that HA can alter, tune, or improve the properties of contact lenses. HA can be entrapped or chemical-bound to the hydrogel to provide increased wettability or resistance to protein deposition. One of the most limiting factors for the wider application of HA is the high cost. Although dilution can reduce the cost, the effectiveness of HA will diminish. Once this issue has been overcome, HA will be commonly incorporated into new lenses.

## 3. Outlook for CL Materials

### 3.1. Drug Delivery

Today, CLs are increasingly seen as a tool for specific ophthalmic treatments that require delivery of a drug to the eye. The low bioavailability (about 5%) of common ophthalmic treatments, such as eye drops, is a concern to delivering effective treatments [168]. In turn, practitioners rely on new lenses to be developed to improve these treatments, which is reliant on material scientists developing new materials. This is evident by the publication of review papers in the last few years [27,28,169]. These reviews contain significant detail on the latest drug delivery mechanisms, materials, and relevant literature of modern drug delivery, including general drug delivery within the body. The majority of the referenced literature is after the year 2000, indicating the significant advances and interest in the field within the last 20 years. In this paper, we highlight the trends in the CL materials used for ocular drug delivery. Some of the methods investigated include drug-loading by nanoparticle- or polymeric encapsulation [170,171,172,173], β-cyclodextrin delivery [40,174,175,176], molecular imprinting [177,178,179], and solution soaking [37,180,181,182]. Molecular imprinting involves fabrication of the hydrogel alongside the drug. Typically, increasing crosslinking and addition of a porogenic solvent facilitates this by creating more cavities for residing the drug [179]. The nanoparticles used in these examples, which were all polymeric, encapsulated the drug. The biocompatible polymers responded to the ocular environment to release the drug. Ethyl cellulose and lactic-co-glycolic acid are two examples [171,172] These are all of scientific interest; however, more should be done to access their practical application. This can include clinical trials or practicality of manufacturing.

**Figure 14:**
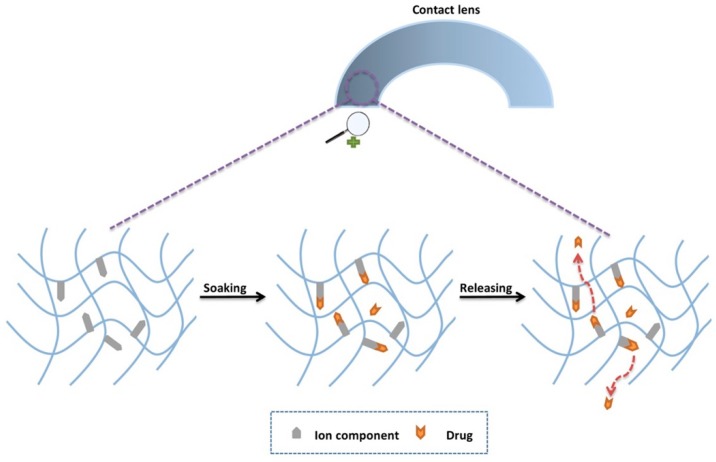
Drug-binding and releasing mechanism for a contact lens with ionic sites within the structure. Reprinted from Journal of Controlled Release, 281, Xu, J.; Xue, Y.; Hu, G.; Lin, T.; Gou, J.; Yin, T.; He. H.; Zhang, Y.; Tang, X. A comprehensive review on contact lens for ophthalmic drug delivery, 97, Copyright 2018, with permission from Elsevier [27].

**Figure 15:**
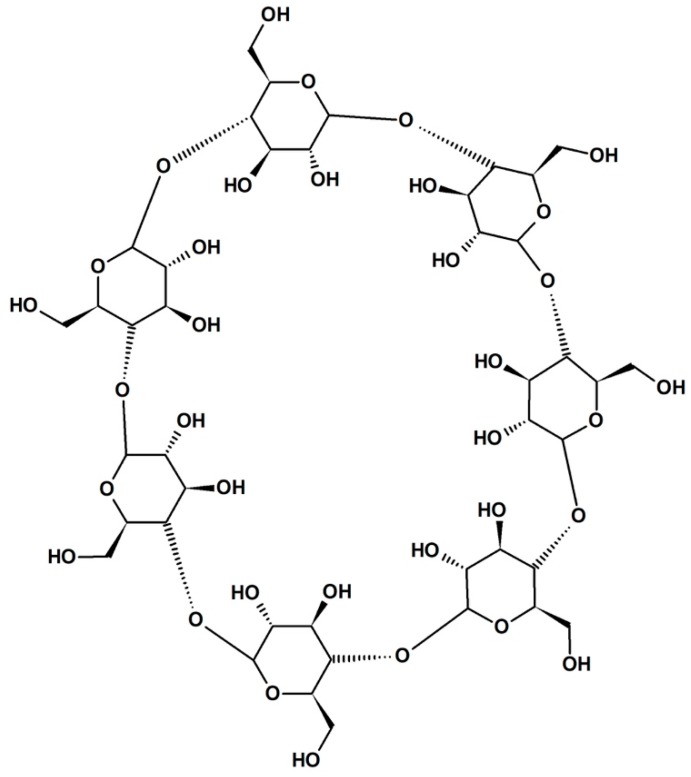
Chemical structure of beta-cyclodextrin. The inner environment of cyclodextrin is hydrophobic, suitable for drug-binding, whereas the outside is hydrophilic, providing suitable bioavailability.

The properties of an incorporated material into CLs can be used as improved drug-binding mechanisms [183]. For example, incorporation of ionic monomers can create binding sites for a polar drug to bind to (Figure 14), meaning the lens can retain the drug until it is placed onto the eye. MAA, for example, is one comonomer commonly used for the formation of hydrogels, but which also has an anionic group to facilitate drug-binding. Another clever use of new materials was the use of pendent- or copolymerized cyclodextrins for introducing both hydrophilic and hydrophobic character to the CL [174,176]. The inside of the 7-unit ring of cyclodextrin is hydrophobic, ideal for drug complexing, whereas the outside of the molecule is hydrophilic to provide the lens with suitable biocompatibility (Figure 15). Dos Santos et al. loaded hydrocortisone and acetazoamide into a HEMA-hydrogel lens, achieving a release profile of several days [174]. An alternative method has involved, using vitamin E to essentially create a barrier to improve the release profile of loaded drugs [25,184,185]. The hydrophobic barrier interacts with the drug, which greatly increases the permeation time rather than simply eluting from the lens. Vitamin E contains a large hydrocarbon side chain and methyl groups to provide the necessary hydrophobic character. In another experiment by Kim et al., vitamin E was easily incorporated into commercial silicone-hydrogel lenses such as ACUVUE^®^, OASYS™, O_2_OPTIX™, and NIGHT&DAY™ [184]. Some of the delivered drugs included cysteamine [186,187] and pirfenidone [188]. Typically, vitamin E was incorporated into the hydrogel through soaking, and the hydrophobic character means it is unlikely to leech out, compared with a hydrophilic component such as PVA. This is a straightforward process that could be adopted into more widespread usage to improve elution times of drugs from CLs. Although, strictly speaking, there was no fundamental change to the hydrogel networks, the work is worthy of inclusion given the profound impact on drug-release profiles. Some examples used straightforward incorporation methods, which could be a manufacturing consideration.

Modern CL materials have shown much-improved drug delivery capacity than current ophthalmic treatments, such as eye drops. Further modification of the base CL polymer structure is required to improve drug delivery. Molecular grafts, particle encapsulation, and soaking are the most common methods for modification. Despite evidence of the improving performance of these materials, more must be done in order to take these materials to the point of commerciality. The biggest barrier to the market is the cost of clinical trials, as well as the manufacturing requirements. Another note of interest is that most of these successful works have been based on HEMA-hydrogels [189]. HEMA-based hydrogels have an abundance of easily accessible chemical functional groups for modification, rather than silicone. In some cases, silicone-based hydrogels were more suitable to host drugs, such as the vitamin E barrier [27]. This represents an interesting position for CL materials, where the requirement of increased hydrophilicity for comfort and oxygen permeability has driven the increased usage of silicone-hydrogels over HEMA-hydrogel lenses. Conventionally, many drugs are chosen based on a strong hydrophobic character [190]. Development of suitable drug-delivery lenses will mostly depend on the intended time of treatment, and the length of wear that facilitates this. In turn, the length of wear will best define what type of CL material should be used and what modification should support the drug release.

### 3.2. Emerging CL Materials Technologies

In this section, we will discuss several emerging materials technologies that could be relevant to future CL materials. Biocompatible materials have become of great interest to the scientific community, particularly as new materials have been innovated [191,192,193,194]. These biocompatible materials could be an avenue for CL researchers to develop novel materials. The necessity for biocompatibility is an obvious reason why these materials could be of interest. Two such areas that have seen particular growth are double-network/interpenetrating hydrogels and pH-responsive polymers. Although some interest has been expressed in areas such as liquid crystal CLs [195,196], the number of research publications remains somewhat limited compared to these other technologies. Briefly, the liquid crystal contact lens is of interest to treating presbyopia, due to the liquid crystal’s on/off character to alter the lens power in the region of +2.00 D. The liquid crystal lens has anisotropic refractive indexes, realigning the molecules when a voltage is applied. Commonly, the lens design often involves PMMA to support the liquid crystal phases [197]. Perhaps the biggest difficulties with these lenses is ensuring ocular health, comfort, and a power source.

**Figure 16:**
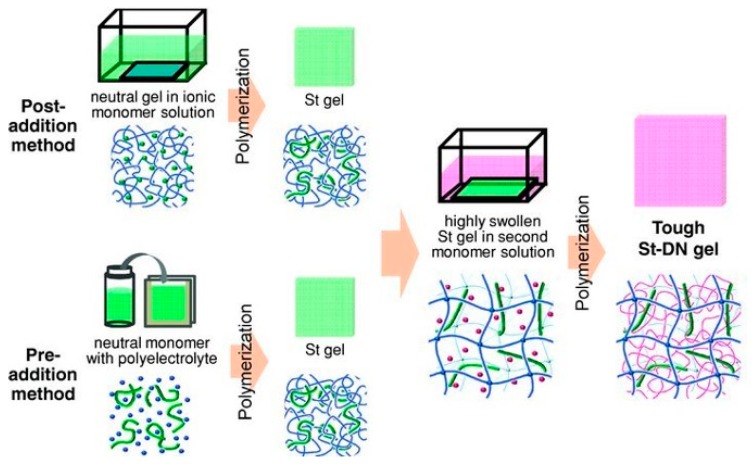
A double-network hydrogel combines the properties of two hydrogels. The gel networks are interpenetrating, meaning they form a new gel that retains the properties of each individual gel. Reprinted with permission from John-Wiley and Sons [199].

Double-network/interpenetrating hydrogels combine two gels by interconnecting the gel networks together, resulting in a new gel composite (Figure 16). Essentially, the network of one gel is intertwined with the other, rather than the formation of a traditional copolymer gel. This is a subtle, but significant, difference—e.g., a double network gel could be formed by two polymers with different functional groups, meaning they could not be copolymerized. Figure 16 also shows that these hydrogels can be formed pre- and post-formation of one gel. Formation of these new materials has been shown to improve the bioavailability/compatibility of hydrogels [198,199]. Some of the materials investigated are commonly known CL- or modification-materials, such as HA, chitosan, thiol-functionalized PEG, poly-2-hydroxyethly acrylate (HEA), and polyacrylamide (PAA). With the large number of materials used in CL synthesis, this method could produce a unique double-network contact lens with improved functionality.

Double-network hydrogel formation is fundamentally a chemical technique to improve the bulk properties of the material. These hydrogels could be manufactured in the same manner as current CL hydrogels, yet enhance the CL properties. This area is of interest to the CL community; a patent was assigned to Stanford University in 2010 for interpenetrating polymer-network contact lenses [200]. One example by Yañez et al. used interpenetrating networks composed of HEMA and polyvinyl pyrrolidone (PVP) to develop new comfortable SCLs [201]. PVP was semi-interpenetrating and leeched from the lens as a comfort mechanism rather than a true double-network hydrogel. These gels have been tailored for applications involving tissue engineering, emphasizing the robustness of the gel [202]. The inner eyelid (conjunctiva) and cornea are two different environments sensitive to the properties of the CL. Castellino et al. combined PDMS-MAA-HEMA to form an interpenetrating hydrogel network [203]. Their purpose was to form a general bioavailable material, rather than a material for specific ophthalmic applications. Wang and Li synthesized a double-network hydrogel of TRIS and HEMA-phosphorylcholine (PC) as a potential ophthalmic material [134]. At higher PC (20%) content, the double-network gels had a low water-contact angle (50°) and a high modulus (7 MPa). These are very attractive qualities for CL hydrogels, which could be further developed. Other double networks of silicone have been investigated for other research fields, suggesting it could be more widely used for CLs [204,205,206]. These works incorporated organic components, which would be essential for ocular biocompatibility. This type of hydrogel could be the next evolution of contact lenses.

**Figure 17:**
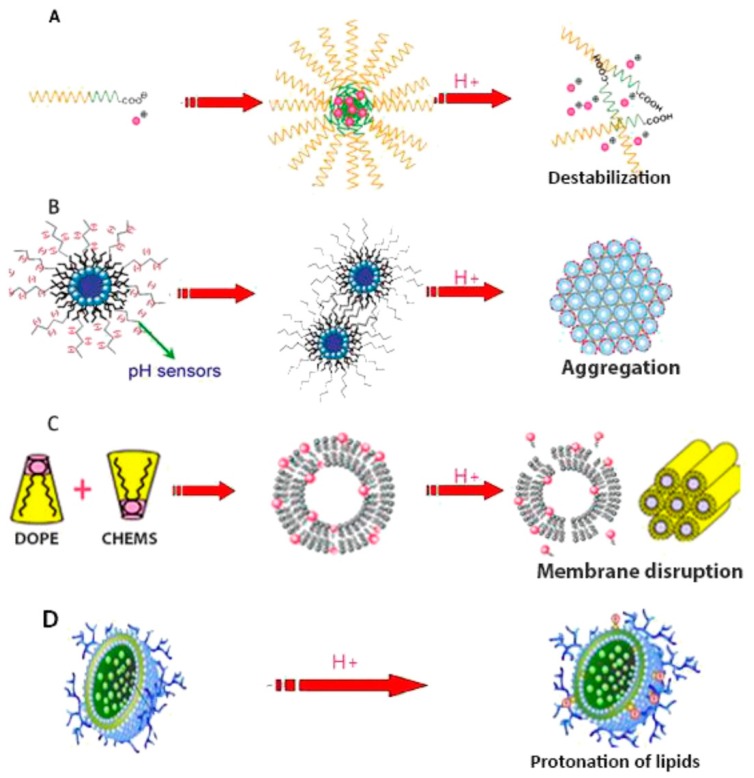
Mechanisms of action for pH-responsive drug-delivery polymers. Reprinted from Biomaterials, 85, Kanamala, M.; Wilson, W.R.; Yang, M.; Palmer, B.D.; Wu, Z. Mechanisms and biomaterials in pH-responsive tumour targeted drug delivery; a review, 152, Copyright 2016, with permission from Elsevier [210].

We have already discussed some of the more popular drug-delivery materials in a previous section. However, there are new concepts within biocompatible hydrogels as potential drug-delivery mechanisms [207,208]. Temperature- and pH-responsive polymers are materials that change their macromolecular conformation when exposed to a specific temperature or acidic/alkaline conditions (hence, pH-responsive) [209]. For pH-responsive polymers, there are four principal mechanisms by which the conformation is changed, and this is summarized in Figure 17. The acidic/alkaline conditions of the body can induce protonation, stripping the coating and conformation changes to the polymer and allowing release of the drug. As such, they are becoming exciting as drug-delivery mechanisms within the human body [210]. The concept was recently investigated for drug-delivery contact lenses [211]. Researchers here used a film of cellulose acetate to deliver betaxolol hydrochloride over a sustained period. They showed that 25% of the drug was delivered to rabbits in the first 3 h of use, and 66% had been delivered within 72 h [211]. The cellulose acetate film was attached to a CL composed of HEMA-NVP-TRIS. Cellulose is a biopolymer and therefore has high biocompatibility, meaning it is suitable for the ocular environment.

Temperature-responsive polymers, when exposed to sufficient heat, undergo conformation changes, which allows the release of the embedded/encapsulated drug. Once the conformation change is complete, the drug will be released in the highly aqueous environment [212,213]. These papers describe in detail the mechanisms of these polymers [212,213]. This could be relevant to contact lenses, as typically there is a significant difference between the storage/room temperature and the eye. A lens composed of such a polymer would undergo a conformation change on insertion to the eye. This temperature difference could be harnessed for drug-delivery CLs. Polyethylene glycol (PEG) has seen growth in this area as a block-copolymer graft to other moieties, such as poly(*N*-isopropylacrylamide) (p(NIPAAm)) and HEMA. Silicone rubbers have even been mentioned as potential drug-delivery vehicles by Fenton et al. [207]. These materials are known for their ocular compatibility, meaning there is design space to be explored for improved drug-delivery contact lenses. Jung et al. studied more common contact materials, such as HEMA, due to the ability to modify conformation with differing conditions, such as nanoparticle loading into the lens [214].

These emerging materials technologies offer interesting new routes for future CL development. We have provided some approaches, such as novel materials in the form of liquid crystals, or evolving the CL material through methods such as double-network hydrogels. In addition, temperature- and pH-responsive polymers are growing drug-delivery vehicles, but have not yet been widely explored for CLs. What is encouraging is the fact that many of these methods involve many of the same materials used for current CLs, meaning adapting to CLs is a clear possibility in the near future. However, despite these encouraging signs, there are significant challenges for the ocular environment, which remain prominent given the aging population worldwide [215]. This includes specific aspects, such as anti-fungal drug delivery, which are not yet ready for commercial specifications [216]. The biggest challenge for these materials is the efforts in other CL research, as previously discussed.

## 4. Manufacturing Perspectives

**Figure 18:**
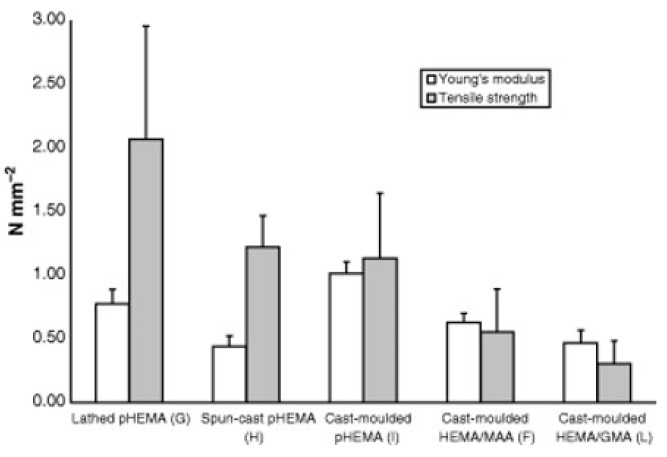
Tensile and Young’s modulus data for HEMA-based hydrogel lenses produced by different manufacturing methods. Reprinted with permission from John-Wiley and Sons [49].

The final part of developing a CL is how the new material will be manufactured. New technologies have emerged to enhance CL properties, such as plasma processing, to introduce hydrophilicity to silicone lenses. CL manufacturing is an ever-evolving area, with manufacturers constantly improving their methods. It is probably fair to say there is much the public does not know about the specific CL manufacturing methodologies. This includes operating conditions, speed of lathes, tip-edge parameters, and exact chemical recipes, amongst a huge number of parameters. For researchers, this can be a difficult area to evaluate, given such lack of information. However, researchers can still analyze the products from manufacturers to help shape the direction of their research. There is literature concerning the differences in the mechanical properties of common HEMA-derived hydrogel lenses based on the manufacturing technique (Figure 18) [49]. The polymerization and manufacturing method were responsible for the differences in the hydrogel properties. Improving the polymerization steps during manufacturing could improve the properties of the lens materials. The manufacturing- or process-driven development is likely pursued for economical or environmental reasons, or both. For example, Nelfilcon A PVA lenses use water as the solvent, which is much easier to handle than organic solvents and does not require further processing [16]. Here, we will discuss some specialist manufacturing techniques seen in other applications of optics that could be used for CL manufacturing. We also discuss some aspects of manufacturing, such as molding, and the materials that have been investigated in recent literature.

Materials are an important consideration for the molding processes, i.e., both cast- and spin-molding. The interaction between the mold surface and polymer solution will affect the surface finish of the CL. Some examples of common molding materials are polypropylene [42] and quartz [16]. Polypropylene is useful due to the ease of handling and high melt temperatures. The polymer is hydrophobic, meaning manufacturers choose this with the intent that the contact lens will not adhere strongly to the mold surface on polymerization (particularly hydrophilic materials). Similarly, quartz (SiO_2_) has non-sticking qualities that are useful for manufacturing lenses. This is a practical consideration to reduce defect occurrence in the final CLs. The recent patent literature has many examples of the new materials being considered as a lens mold material [217,218,219,220]. These patents are assigned to CLs manufacturers, showing the commercial implications of improving this aspect of manufacturing. Some of the mold materials include alicyclic polymers or polyoxymethylene, which are non-polar and polar, respectively. The mold material can influence the adhesion to the mold, which, in turn, could influence the resulting wettability properties of the hydrogels.

Ultra-precision- and nano-manufacturing is an area of research that could be applied to CLs to enhance the physical and optical properties of the material [221,222,223]. These techniques have been successfully implemented in a number of areas, including biomedical device manufacturing, optics, and more [224]. Ultra-precision lathes are now used in the production of specialty CLs to obtain nanometer control of the surface, which can control the wettability characteristics of polymers [225] and glass surfaces [226]. However, to date, this has not been extensively applied to contact lenses, or at least cannot be seen in the published literature. However, the control of wettability is possible by using machining techniques [227]. Freeform optics are a special type of optical lenses with irregular contouring on a non-symmetrical surface. Improvements in optical performance have seen their use in applications relating to imaging, illumination, and aerospace interests [228,229]. This is an increasingly important area of precision manufacturing, requiring consideration of the design, as well as machining and evaluation aspects [230]. Furthermore, servo-control machining could be feasible to fabricate freeform optical CLs for specialty applications, such as myopia control. Fast servo-manufacturing of freeform optics has even been shown to be feasible on polymers such as polystyrene [231]. This suggests that the use of a similar technique could be employed on a pre-hydrated CL material. The usage of such precision manufacturing techniques to intraocular lens manufacturing was also reported [232]. All of these techniques are subject to the effect which the resulting material will have on the ocular environment, such as the effect on the tear film, protein deposition, optical properties, etc. Finally, there is a cost component to manufacturing in a commercial space that would also need to be considered.

## 5. Conclusions

The purpose of this review was to bring together state-of-the-art research on contact lenses and their modification. Modern CL research aims to better deliver ophthalmic treatments or improve existing issues with contact lenses. Overall, modern CL materials are an evolution of the well-known lens materials based on HEMA and silicone hydrogels. These hydrogels grafted or incorporated various bioavailable components, such as PEG, HA, chitosan, β-cyclodextrin, cellulose, and other moieties. These biomaterials are very biocompatible due to their inherent chemistry. The biomaterials field is exciting and dynamic, where new and improved biocompatible, drug-delivering materials are constantly being developed. One of the biggest hurdles to overcome is the cost and practicality of incorporating such species to a new lens. We highlighted some emerging fields related to CLs due to the materials involved or which have only recently been applied to CLs. The application of new manufacturing methods can offer other solutions to specific challenges for CLs: new mold materials, surface contouring, etc. CL materials are now resilient and modifiable, and are thus suitable for these improving manufacturing processes. Advances in precision manufacturing, such as the production of freeform optics or modification of surface properties to improve wettability could be applied to future CLs. In summary, future CL materials will continue to push the boundaries of the biocompatibility and materials sciences to better tailor to the needs of a growing CL-using population.

## Figures and Tables

**Table 1:** General pros and cons of current CL material classes.

CL Material	Pros	Cons
PMMA	Inexpensive, well-understood polymer	No oxygen permeability, inflexible on the eye
RGP	High oxygen permeability, durable	Expensive regents, requires hydrophilic comonomer, can be abrasive
HEMA hydrogel	Inexpensive, biocompatible, abundant copolymer possibilities	Low oxygen permeability, protein deposition issues
Silicone hydrogel	High oxygen permeability, durable, comfortable	Expensive regents, requires hydrophilic comonomer, can be abrasive
PVA	Inexpensive, straight-forward manufacturing, biocompatible	Low oxygen permeability, fixed water content

**Table 2:** Generalized properties of some common CL materials.

Material	Oxygen Permeability (Dk/t)	Water Content (wt %)	Modulus (MPa)	Wear Time (days) *
PMMA	0	0	1000	<1
PMMA-silicone	15	0		
Silicone-HEMA (rigid)	10–100	0	10	
HEMA hydrogel	10–50	30–80	0.2–2	1–7
HEMA-NVP				
HEMA-MMA				
Silicone (PDMS) hydrogel	60–200	20–55	0.2–2	~7–28
TRIS-DMA				
PDMS-HEMA				
PVA	10–30	60–70		<1

* Maximum wear time without extensive complications to the eye before lens disposal.
